# The complex portal - an encyclopaedia of macromolecular complexes

**DOI:** 10.1093/nar/gku975

**Published:** 2014-10-13

**Authors:** Birgit H.M. Meldal, Oscar Forner-Martinez, Maria C. Costanzo, Jose Dana, Janos Demeter, Marine Dumousseau, Selina S. Dwight, Anna Gaulton, Luana Licata, Anna N. Melidoni, Sylvie Ricard-Blum, Bernd Roechert, Marek S. Skyzypek, Manu Tiwari, Sameer Velankar, Edith D. Wong, Henning Hermjakob, Sandra Orchard

**Affiliations:** 1 European Bioinformatics Institute (EMBL-EBI), European Molecular Biology Laboratory, Wellcome Trust Genome Campus, Hinxton, Cambridgeshire CB10 1SD, United Kingdom; 2 Department of Genetics, Stanford University School of Medicine, Stanford, CA 94305–5477, USA; 3 Department of Biology, University of Rome, Tor Vergata, Rome 00133, Italy; 4 UMR 5086 CNRS, Université Lyon1, Institut de Biologie et Chimie des Protéines, 7 passage du Vercors, 69367 Lyon Cedex 07, France; 5 Swiss-Prot Group, SIB Swiss Institute of Bioinformatics, Centre Medical Universitaire, Geneva, Switzerland; 6 Stammzellbiologie, Institut für Anatomie und Zellbiologie, GZMB Universitätsmedizin Göttingen, Ernst-Caspari-Haus, Justus-von-Liebig-Weg 11, 37077 Göttingen, Germany

## Abstract

The IntAct molecular interaction database has created a new, free, open-source, manually curated resource, the Complex Portal (www.ebi.ac.uk/intact/complex), through which protein complexes from major model organisms are being collated and made available for search, viewing and download. It has been built in close collaboration with other bioinformatics services and populated with data from ChEMBL, MatrixDB, PDBe, Reactome and UniProtKB. Each entry contains information about the participating molecules (including small molecules and nucleic acids), their stoichiometry, topology and structural assembly. Complexes are annotated with details about their function, properties and complex-specific Gene Ontology (GO) terms. Consistent nomenclature is used throughout the resource with systematic names, recommended names and a list of synonyms all provided. The use of the Evidence Code Ontology allows us to indicate for which entries direct experimental evidence is available or if the complex has been inferred based on homology or orthology. The data are searchable using standard identifiers, such as UniProt, ChEBI and GO IDs, protein, gene and complex names or synonyms. This reference resource will be maintained and grow to encompass an increasing number of organisms. Input from groups and individuals with specific areas of expertise is welcome.

## INTRODUCTION

Biological processes are driven by the interactions of proteins with other molecules in an organism. These interactions may be transient (e.g. signalling receptor–ligand interactions) or lead to the formation of stable biological complexes (e.g. minichromosome maintenance complex, EBI-913604). Many molecules exist only in an obligate complex formation (e.g. collagen type I, EBI-2325312). Many databases deal with protein, small molecule and polysaccharide functions, for example, UniProtKB (1), Gene Ontology (GO) (2), ChEBI (3), ChEMBL (4) and their interactions (e.g. IntAct (5), MINT (Molecular INteraction Database (5,6)), MatrixDB (7) or DIP (Database of Interacting Proteins (8)) but no central, integrated resource has so far been available to hold this type of information for stable, macromolecular complexes. The Complex Portal, hosted by the IntAct team at the European Bioinformatics Institute (EBI) (www.ebi.ac.uk/intact/complex), provides a framework for the capture of such data, and the existence of web-based editorial tools not only enables collaborating groups to capture standards-compliant experimental data (5) but also to contribute to the curation of the growing collection of manually curated protein complexes. Several databases (such as CORUM (9)) exist that have previously collated information on protein complexes but they provide limited data integration across resources and have tended to concentrate on a more limited species range. UniProtKB/Swiss-Prot contains a wealth of data pertaining to the behaviour of individual proteins within larger complexes in the form of free-text statements. Curators from the UniProt Consortium collaborate with the Complex Portal to help extract and structure these data. The Protein Ontology (PRO) provides an ontological representation of protein-related entities by explicitly defining them and describing their relationship (10). This includes the existence of proteins within larger macromolecular complexes. The PRO consortium, by collaborating with the IntAct group, will contribute to both extend the number of complexes which are available as objects within the PRO hierarchy while making more richly annotated data on these entities available in a broader range of formats through the Complex Portal. Model organism groups, such as *Saccharomyces* Genome Database (11) and WormBase (12), are contributing expertise and curation effort within their organism of interest, while the MINT database is augmenting the resource with additional expertise on the composition of protein complexes. Curators at the EBI ensure that entries relating to conserved complexes remain consistent across multiple species, or that differences that have evolved across a taxonomic range are suitably highlighted. Long-term data maintenance, such as regular updates of controlled vocabulary terms and underlying protein sequence data, are also provided by the IntAct team.

Entries combine biological information about the molecular function, biological processes, cellular location and pathways of a complex with information about its constituents (proteins, nucleic acids, small molecules and polysaccharides), topology, stoichiometry and structural assembly. Structured information about complex ligands, diseases they are involved in and drugs they act as targets for is also added to appropriate entries. The use of the Evidence Code Ontology (13) allows us to indicate for which entries direct experimental evidence is available in a molecular interaction databases, such as IntAct (www.ebi.ac.uk/intact), or if the complex has been modelled from more limited evidence, or inferred from component sequence homology or orthology.

## CONTENT

### Coverage

The Complex Portal contains complexes from more than 10 model organisms, with a current emphasis on human, mouse, *Saccharomyces cerevisiae* and *Escherichia coli*. The working definition of a stable protein complex is an entity that contains two or more macromolecules that can be co-purified and for which there is evidence (experimental or inferred) that these molecules interact with each other and have a demonstrated biological or MF. The definition currently specifically excludes more transient interactions, such as enzyme-substrate and receptor-ligand complexes, unless the binding of the substrate or ligand is required for complex formation to occur, e.g. PDGF receptors only dimerize when the ligand binds so the tetrameric receptor-ligand complex has been curated (EBI-9080360), in contrast to the dimeric receptor which does not exist *in vivo*. Nucleic acids are only included in the list of participants if they are integral to the formation of the complex (e.g. DnaA-DNA complex, EBI-6552438). Similarly, small molecules, such as cofactors, are captured when stably bound into complexes. Homology modelling is only allowed across closely related species (e.g. mammals), as one use case for this resource is to enable the study of the evolution of protein complexes and highlight examples where function but not subunit content may have been conserved, or vice versa. As indicated above, modelled or inferred complexes are clearly marked using the evidence ontology terms and may be filtered out from experimentally verified data using a simple query.

### Quality control

As with IntAct and many other manually curated databases, each entry is checked by a second curator before it is released, enabling the provision of highly accurate and up-to-date information. However, no data set can claim to be totally error-free and new information is constantly being published. Therefore, the user is encouraged to feedback, or request curation of a specific complex via the <Feedback> button on the website or by directly emailing intact-help@ebi.ac.uk.

### Search

The portal query interface allows users to search the complex information based on the identifier, name or synonyms of the complex or of the individual components (proteins, nucleic acid or small molecules) present in the complex as well as cross-references and species of complexes. Users can search for a single term or a mix of terms. The latter can be of the same or different kind, such as a single UniProt ACs, a list of mixed protein and gene identifiers, a complex name and its species or a GO ID (e.g. GO:0016491). The use of wildcards (‘?’ and ‘*’) has been enabled, e.g. for a search of isoforms of ‘Q07817’ search with ‘Q07817*’. Blanks in search strings are by default interpreted as ‘OR’. For more precise searches use the Boolean operators ‘AND’ and ‘NOT’, use prepends ‘+’ (= AND) or ‘-’ (= NOT), or use parentheses () or double quotes (“) to specifically separate or combine certain terms (e.g. <‘nuclear pore’> is more precise than <nuclear AND pore> which is more precise than <nuclear pore> as the latter equals <nuclear OR pore>). More refined searches can be performed using the Complex Query Language, CQL. CQL has been specifically designed for searching complexes but has retained those fields that overlap with the Molecular Interactions Query Language, MIQL, such as ID, alias, species, biological role and feature type. More detail on the search options can be found at http://www.ebi.ac.uk/intact/complex/help/. The search results can either be filtered or a particular entry can be selected in order to view its details. Currently, it is possible to filter by species, and by biological role (e.g. enzyme) and interactor type (e.g. protein) as defined using the Proteomics Standards Initiative - Molecular Interactions Controlled Vocabulary (PSI-MI CV) (14).

The manual curation of the complexes using hierarchical ontologies, such as the GO or the National Center for Biotechnology Information (NCBI) taxonomy (15), also allows queries based on these hierarchies. For example, it is possible to pull out all enzyme complexes by searching on the parent term ‘catalytic activity’ (GO:0003824, Figure 1) which will find all examples within the current collection, although each has been more specifically annotated to an appropriate child term. Similarly, it is possible to find all mammalian complexes by using the search term ‘species:40674’ (mammalia).

**Figure 1.**
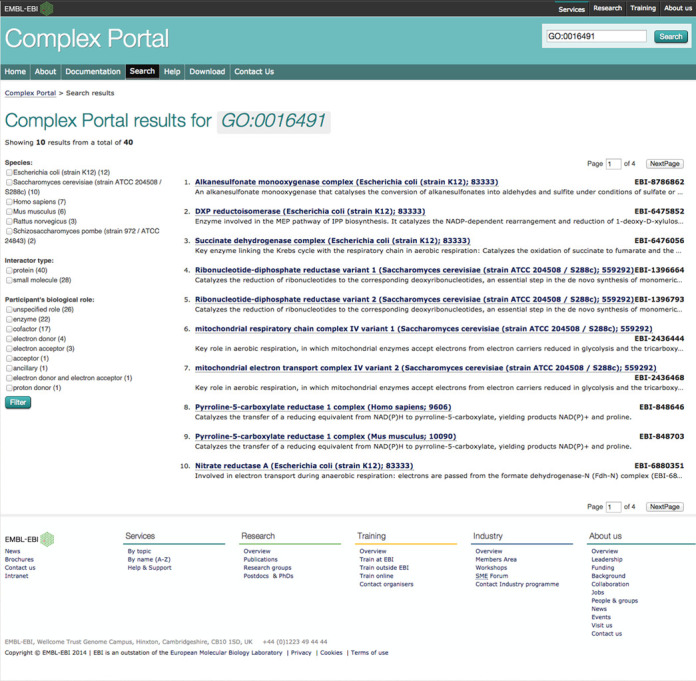
Screenshot of the results for the GO search term ‘GO:0003824’ (catalytic activity). Search results are displayed as 10 per page and filters for refining the search are shown in the left-hand side panel. The number of hits for each filter option is given in parenthesis.

### Complex details

**Figure 2.**
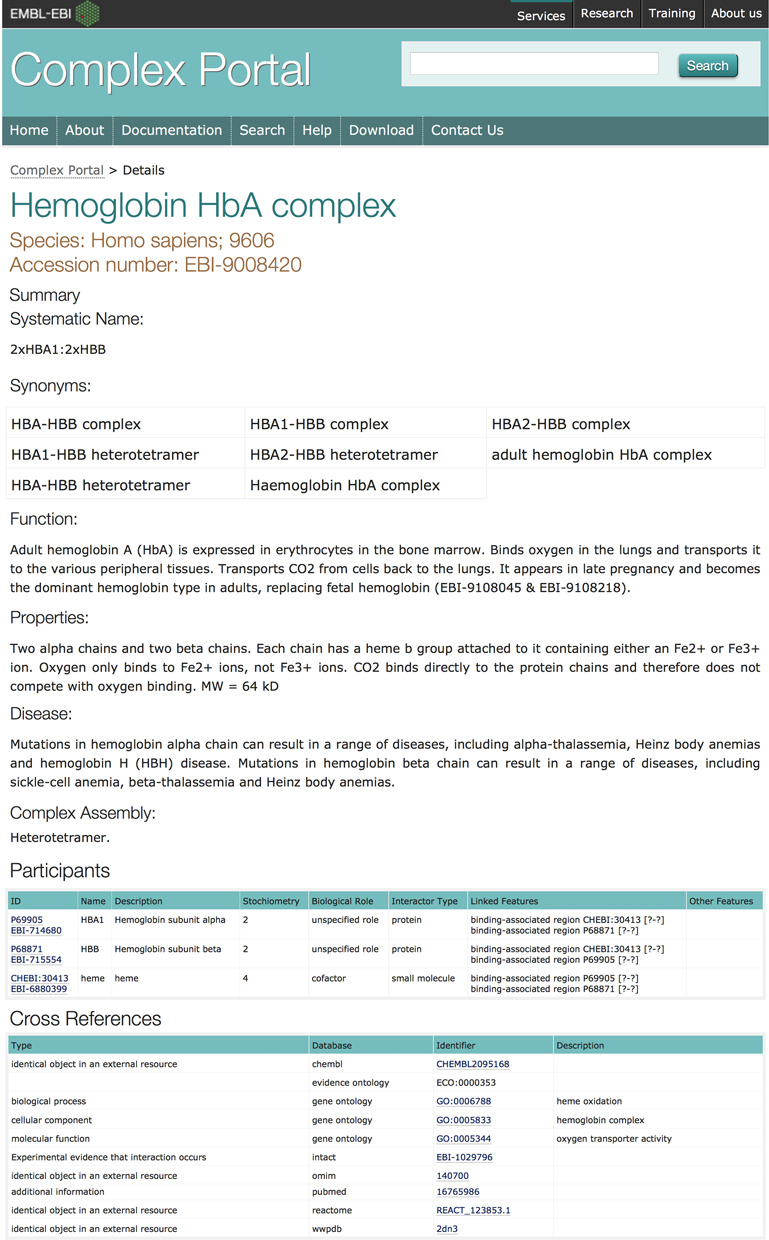
Screenshot of the details page of the human haemoglobin HbA complex (EBI-9008420). The Cross References table has been shortened to fit in the figure. Clicking on the hyperlinks will open the details directly in the external database.

Each complex has as a minimum a unique accession number, a recommended name, a list of participants, a functional description and a list of cross-references (Figure 2).

Complexes are curated based either on direct evidence in the literature, or inferred from closely related species (e.g. mouse PCNA, EBI-1208675) or homologous complexes in the same species, for which experimental evidence exists (rat hemoglobin variants, EBI-9105417 and EBI-9105480), or based on background scientific knowledge of an expert submitter (e.g. existence based on pharmacological evidence, e.g. GABA receptors, EBI-9008426). The type of evidence that exists for each complex entry is identified by using Evidence Code Ontology (ECO) codes. For ECO codes currently in use please refer to the online documentation (http://www.ebi.ac.uk/intact/complex/documentation/). In cases where the existence of a complex has been inferred by sequence homology of the components, rather than from direct experimental evidence, the details of the experimentally verified complex, on which it has been modelled, are given. These entries will be updated when experimental evidence becomes available.

Complex members are identified via unique accession numbers with direct links to the respective databases (UniProtKB for proteins, ChEBI for small molecules), name and description of the molecule (imported from the above reference databases), stoichiometry (when known), interactor type and the biological role of that molecule within the complex. Known protein binding regions are identified by their start and end residue numbers and cross-referenced to InterPro (16) if they match a functional domain (EBI-9519429), whereas unknown regions are identified by range ‘?-?’ (EBI-706546). The linking of binding regions enables the systematic capture of the topology of a complex in a format which will subsequently enable the automated derivation of a graphical representation of this topology. Other features, such as post-translational modifications and disulphide bridges, which are required for binding, are also listed (e.g. EBI-8869931).

Each complex has up to three name categories: firstly, a mandatory recommended name that is either taken from the GO or is the most commonly used name in the literature. In parallel, missing terms are added to the GO and the protein complex hierarchy within the GO is being revised and augmented. Secondly, additional names for the complex are added to the list of *synonyms*. Thirdly, in collaboration with Reactome (17,18), we give each complex a *systematic name*, which is essentially a string of gene names of the complex participants, separated with a colon (:) and stoichiometry indicated, e.g., 2xHBA1:2xHBB for haemoglobin HbA. For further details of the Reactome nomenclature rules, see (19).

Further information on the complex is added by the curator providing a detailed description of its *function*, and any processes it is involved in, as well as information about *properties*, such as molecular weight, complex size or internal topology of the complex, where available. Complexes with complete stoichiometry are also annotated with the relevant *assembly* terms, such as homodimer or heterotrimer. Where applicable, important *ligands* are listed and short descriptions are given about *diseases* with which the complex is associated.

To aid data integration and to provide maximum additional information to the user, related classes or instances of the complex in other databases are provided as cross-referenced links. These may include links to ChEMBL, EMDataBank (20), the GO, MatrixDB, PDBe (21) and Reactome. Links to experimental evidence proving the existence and content of a particular complex are provided as IMEx (International Molecular Exchange Consortium (22)), IntAct, MINT, MatrixDB or DIP cross-references and, as indicated, ECO codes are used to further indicate the level of evidence available for the complex. For enzymes we provide a link to the Integrated relational Enzyme database (IntEnz (23)). When a complex is involved in a disease we provide cross-references to Online Mendelian Inheritance in Man (OMIM, http://omim.org/), Experimental Factor Ontology (EFO) and/or Orphanet (http://www.ebi.ac.uk/efo/about.html and http://www.orphadata.org/cgi-bin/inc/ordo_orphanet.inc.php). As EFO is in the process of compiling a new disease ontology we will progressively be cross-referencing to EFO/Orphanet/ORDO. Further information in the literature on the complex, such as an appropriate review or a functional study, is cross-referenced to Europe PubMed Central (24).

All complexes are annotated to GO terms. Every complex is annotated to at least one cellular component term and to relevant molecular function and biological process terms for the complex as a whole, rather than annotation of individual components as is the focus of other annotation efforts. New terms are created as appropriate.

## WEB APPLICATION, WEB SERVICE AND DOWNLOAD OPTIONS

A new index has been built and a public RESTful web service has been provided (http://www.ebi.ac.uk/intact/complex-ws/) for programmatic retrieval of complexes. The current webservice provides only two methods but will be expanded as new features are added. A method ‘/search’ will take as parameter for any CQL query and return a custom JSON which only gives a summary of the existing complexes hitting the user query (complex name, species, description and internal identifier). The user can then get the full details of the complex of interest using the ‘/details’ method which returns another custom JSON describing the complex cross-references, participants, etc.

The web application has been built on top of the RESTful service for searching and viewing of individual complexes.

Files can be downloaded in PSI-MI XML2.5 format from our dedicated ftp site: http://www.ebi.ac.uk/intact/complex/download/.

## COLLABORATIONS AND COMMUNITY INVOLVEMENT

The Complex Portal has been designed to provide a framework in which groups are able to contribute their expert knowledge by annotating complexes within a particular defined area. The IntAct molecular interaction database is responsible for training, data quality, data consistency and long-term data maintenance. While coverage is limited at this early stage of the Portal development, the involvement of multiple contributing groups ensures that this will grow rapidly. We hope that further groups will add to the community effort in the near future, in particular, participation of the plant community would assist in filing this gap in the taxonomic coverage.

For suggestions for new complexes to be added to the database and direct submissions please contact us via the <Feedback> link on the homepage or by directly emailing at intact-help@ebi.ac.uk.

## FUTURE PLANS

Work is continually ongoing to improve the website, search capabilities and download options. The PSI-MI are currently actively working to provide an improved XML format, PSI-MI XML3.0, for the capture of abstracted data, such as protein complexes. Individual complex download files (in both XML2.5 and XML3.0) will be available from both the list of initial search results and the details page. The filter options will be expanded, for example, to make it easier to extract the experimentally verified complexes from those which have been modelled or inferred by homology.

Visualization of complex topology is critical and a complex viewer is currently under development that will display the complex participants and its binding features in a flexible, interactive mode from the information supplied in the underlying XML file. The complex viewer will be wrapped in a BioJs (25) component which will be available in the BioJs registry so it can be re-used by other developers wishing to import and display some or all of these protein complexes, or indeed other data presented in the PSI-MI XML format. Other BioJS components, such as a structural viewer, for those complexes which have been fully, or partially, crystallized and patterns of co-expression data for complex components, will also be incorporated. Ongoing activities include improvements to associated controlled vocabularies and further cross-referencing in addition to increasing the content of the Complex Portal.

We constantly try to improve our databases and services in terms of accuracy and representation and actively encourage user feedback. Please contact the IntAct group if you have any questions via the <Feedback> link on the homepage or email us directly at intact-help@ebi.ac.uk. Information about curation is provided at http://www.ebi.ac.uk/intact/complex/documentation/. Extensive documentation and training material on how to best use our resource is available at http://www.ebi.ac.uk/training/networks. Curation groups interested in capturing macromolecular complex data or experimental interaction data who would like access to the editorial tool are encouraged to contact IntAct to discuss this further (intact-help@ebi.ac.uk).
